# The Evaluation of Ureteroscopy and Pneumatic Lithotripsy Results in Pregnant Women With Urethral Calculi

**DOI:** 10.5812/numonthly.10726

**Published:** 2013-08-12

**Authors:** Maliheh Keshvari Shirvan, Mohammad Reza Darabi Mahboub, Hamid Reza Rahimi, Ali Seyedi

**Affiliations:** 1 Department of Urology, Imam Reza Academic Hospital, Mashhad University of Medical Sciences, Mashhad, IR Iran; 2 Department of Modern Sciences and Technologies, Faculty of Medicine, Mashhad University of Medical Sciences, Mashhad, IR Iran

**Keywords:** Urinary Calculi, Urolithiasis, Lithotripsy

## Abstract

**Background:**

Urinary stone incidence in pregnancy has been reported in a wide range, from 1 in 200 to 1 in 2000 cases.

**Objectives:**

The aim of this study was to investigate the efficacy and safety of ureteroscopic treatment and its results and complications for pregnant women with urinary stones.

**Patients and Methods:**

From 2003 till 2011, 113 pregnant patients with symptomatic urolithiasis were admitted to the urology emergency clinic at Imam Reza hospital. All patients were initially treated conservatively, resulting in spontaneous passage of the calculi in 69 patients. Forty-four patients with symptomatic urolithiasis were included in the study. Post-operative follow ups, including maternal and fetal health was performed by a gynecologist consult fetal heart rate assessment and urine analysis and culture and renal and urethral ultrasonography.

**Results:**

The mean age of the patients was 23 years ± 2 (19-34) and the mean gestational age was 24 ± 3 weeks. The overall and pneumatic lithotripsy success rate was 100%. All patients from the interventional group delivered at term with no fetal or maternal complications. There was no morbidity during and after the operation.

**Conclusions:**

In conditions, medical management of urinary stones and consequent renal colic in pregnant women cannot improve patients’ symptoms, choosing of a surgical method like setting of a DJ catheter or URS and pneumatic lithotripsy can be a safe and effective way for the health of both the mother and fetus. Of course, more research is needed to establish this approach as the standard method in pregnancy urinary stones.

## 1. Background

Urinary stone incidence during pregnancy has been reported in a wide range, from 1 in 200 to 1 in 2000 pregnancies, which is not different from non-pregnant patients (1) and the treatment of symptomatic urolithiasis during the pregnancy period is a challenging issue (1, 2).

Medical management is the first option of treatment in pregnancy; however there are different methods of treating renal stones such as extracorporeal shock wave lithotripsy (ESWL), percutaneous nephrolithotripsy (PNL) and ureteroscopy (URS) and lithotripsy. More than 90% of pregnant women with urinary calculi respond to conservative treatment including IV fluids, prophylactic antibiotics and analgesia, and they can get definitive therapy, post-partum (3). In cases with resistant pain, a surgical option for definitive treatment of the stone must be chosen. Some of the interventions cannot be selected because of the possibility of side effects for the fetus (4) including ESWL which can increase the risk of spontaneous abortion and hearing difficulties (3).

The first study regarding the safety of URS in pregnancy was performed during 1998 (5), and ever since other research have been reported. Some treatment options such as nephrostomy drainage or ureteral stent placement are associated with complications like infection, obstruction, drain dislodgement and are not definitive treatments (6). There is no difference in the complications of URS among pregnant and non-pregnant patients (7) and lithotripsy can be performed with intracorporeal pneumatic lithotripsy and holmium laser lithotripsy which are the most efficient methods of treatment during pregnancy (8-10). There is no difference in the complications of URS among pregnant and non-pregnant patients (7).

## 2. Objectives

In this study, the effects of ureteroscopy and pneumatic lithotripsy on pregnant women with urinary stones have been evaluated.

## 3. Patients and Methods

From June 2003 till April 2011, 113 pregnant patients with symptomatic urolithiasis attended the urology emergency clinic at Imam Reza hospital. All patients were initially treated conservatively, resulting in spontaneous passage of the calculi in 69 patients. 44 patients with persistent symptoms of urolithiasis were included in the study. The study was conducted in accordance with the principles the declaration of Helsinki, 1996 version and good clinical practice standards. The study protocol, informed-consent form, and other study related documents were reviewed and approved by human research ethics committee of Mashhad university of medical sciences. All patients were able to read, understand, and sign the informed consent of the study. An inclusion criterion was having urinary stone-induced renal colic, which had not responded to conservative therapy. Exclusion criteria were the lack of patient’s consent and failure to follow up the patient for any reason. Urine analysis and culture and renal ultrasonography or one shot IVP had been used for symptomatic patients and stone detection.

All patients were admitted and anesthetic assessment was performed. 1 hour before the operation, 1 gram of Cephazolin was injected. Under general anesthesia, ureteroscopy was performed using semirigid 8 fr Karl storz ureteroscope with a guide wire. Also Pneumatic lithotripsy (Swiss lithoclast) and 2.4 fr of long probe was used for 16 patients. A gynecologist consultant performed post-operative follow-ups including maternal and fetal health; fetal heart rate assessment, urine analysis and culture and renal ultrasonography were performed.

## 4. Results

The mean age of the patients was 23 ± 2 (19-34) and the mean age of gestation was 24 ± 3 weeks (12-36). The patients’ characteristics, complaints and the method of diagnosis are shown in Table 1. 

**Table 1.** Trimester, Symptoms, U/A, U/C and Radiologic Imaging, Stone Location, Method of Treatment

Variable	Number of Patients
**Trimester**	
First	2
Second	26
Third	16
**Symptom**	
Pain	44
Gross hematuria	4
Fever and chill	6
**Urine analysis and culture**	
Hematuria	40
Pyuria	36
Positive culture	6
**Radiologic imaging**	
Ultrasonography	44
One shot IVP	2
**Stone location**	
Proximal ureter	2
Middle ureter	10
Distal ureter	36
Left side	14
Right side	26
Bilateral	4
**Treatments used for patients**	
Unilateral	
Lithotripsy	30
Grasper	8
Double J	2
Bilateral	
Bilateral lithotripsy	2
Left lithotripsy and right double J	2

In 4 patients (2 patients with unilateral and the 2 patients with bilateral urolithiasis on the right side) calculi were pushed back and double J catheter was inserted. In 16 patients, the stones were removed using a grasper. In 30 patients with unilateral stones and 4 patients with bilateral urethral stones (2 patient on both sides and 2 on the left side) lithotripsy was performed successfully. Type of intervention is shown in Table 1. 

The success rate of Pneumatic lithotripsy for patients, in whom the stone were not pushed back, was 100%. The mean time of operation for each ureter was 18 minutes. There were no urologic and anesthetic complications during and after the procedures.

All patients in the study delivered at term with no fetal or maternal complications. During the 5 years of follow up, all children were normal in terms of mental and physical growth.

## 5. Discussion

There are different methods for the diagnosis of pathologic hydronephrosis in pregnancy. In depth investigations such as Micelyte and his colleagues' study, ultrasonography was the method of choice for diagnosis (11). Of course its sensitivity in the detection of ureteral stones is restricted especially in the 3rd trimester because of the largeness of the embryonic skeleton. Evans and his colleagues used abdominal X-ray, retrograde pylography and one shut IVP as the 2nd line of diagnosis which had no significant complications (12). In another study, Grenier and his co-workers used color-Doppler sonography for determining pressure location on the ureter against vessels and differentiation of these two (13). In the present study ultrasonography was used as the method of choice for detecting ureteral stones except for 2 patients (4.5%) that were at the 3rd trimester of pregnancy and one shut IVP was performed after ultrasonography.

In a study by Juan, 55.5% of the entire stones were reported to be at the 3rd trimester of pregnancy. Maximum incidence of urinary stones in other series has also been reported to be at the 2nd and 3rd trimesters of pregnancy (11, 14-17). In the present study, 2 case (4.5%), 26 patients (60%) and 16 patients (36.5%) were in the 1st, 2nd and 3rd trimester of pregnancy, respectively. Juan and Lifshitz showed that a significant difference does not exist between left and right side stones (14, 18).

In this study, the stone locations were at the right ureters in 26 patients, the left ureters in 14 patients and bilateral in only 4 patients. The stone was located in the distal ureter in 36 patients, middle part in 10 cases and proximal ureter in 2 patients.

Flank pain was the chief complaint of the patients similar to other series (14, 16-22). However, 60% of patients in Hendricks study complained of lower urinary tract symptoms (LUTS) and urinary infection (23). In the present study, 6 cases (13.5%) had urinary infection (4 patients (9%) pyelonephritis and 2 patients’ cystitis). No signs of azotemia were detected. From the literature, 50-80% of stones during the pregnancy period would respond to conservative treatment and about 1/3 of them required surgical intervention (1, 3, 18, 22).

In a large study in Lithuania on 216 pregnant women with complicated hydronephrosis between 1992 to 2001, supportive treatment including hydration, prescription of spasmo-lytico-analgesic medications and antibiotics were effective in 57% of patients while 43% needed surgical interventions (contriving of a ureteral catheter in 41% and open or percutaneous nephrostomy in 2%) (11). In the current study, among 113 pregnant patients with symptomatic urinary stones, 69 cases passed the stone spontaneously with a pain killer and hydration therapy.

Physiological hydronephrosis is seen in more than 80% during pregnancy period (24), so there is some conflict in differentiating of nephrolithiasis from physiological hydronephrosis in this period (8). In this study hydronephrosis, with or without exposing of the stone, besides the signs and symptoms of nephrolithiasis and positive laboratory tests such as hematuria and pyuria were considered as the pre-operative diagnostic method.

The most common interventional method for symptomatic urinary stones during the pregnancy period is installing a retrograde catheter which is possible by cystoscopy and under local anesthesia. This method is advantageous because of its simplicity, having no radiation and no risk for both the mother and fetus.

In Jarrard’s study, retrograde ureteral catheter by local anesthesia and intravenous sedation was the treatment of choice for pregnancy stones (22). Moreover, in Hendricks study, the insertion of ureteral catheter was the first line of treatment (23).

In the present study, the insertion of DJ ureteral catheter was done for 7 patients (16%). The other therapeutic method is percutaneous nephrostomy which can be done by local anesthesia and under ultrasonographic guidance to decrease complications (18). This method in addition to proper antibiotic coverage can be more effective especially in cases with pyonephrosis. In Van Sonnenbreg’s study, percutaneous nephrostomy with ultrasonographic guidance was performed for 5 pregnant patients with pyonephrosis and even a patient with pain and azotemia who had a history of kidney graft (25). However, using this method in the early stages of pregnancy (before week 22) is accompanied by problems such as repeated catheter obstruction, which needs repeated catheter washing and even replacement. With the development of new ureteroscopic technology and easy intra-abdominal lithotripsy, definitive therapy of urinary stones in pregnancy has recently been taken into consideration.

Ureteroscopy is a usable and safe method. Flexible ureteroscopy has brought the ability to pass a tortuous ureter with a lower risk of perforation. In Lifshitz’s series, rigid or flexible ureteroscopy is presented as the treatment of choice and the first option for symptomatic stones in pregnancy. Cystoscopic installation of DJ ureteral catheter was limited to some special cases like late stages of pregnancy, difficult ureteroscopy and severe urinary infections (14). In another study Juan has successfully done a ureteroscopic lithotripsy under epidural anesthesia (18). This method is well tolerated and different studies have shown no complications in pregnancy. In the current study, lithotripsy was performed for 34 patients (77.5%) with 8 Fr. Semi rigid Swiss pneumatic lithoclasts.

In many different studies, usages of pneumatic lithotripsy and also Holmium-Yag laser have been shown to be risk free and had complications during the pregnancy period but ultrasonic lithotripsy is contraindicated in pregnancy because of a probable embryonic damage. Akpinar used Holmium laser for lithotripsy of ureteral stones in pregnancy during a 5-year period and found it to be a safe and reliable therapeutic option for pregnant women. They also suggest using of a ureteral catheter at least for 72 hours after intra-abdominal lithotripsy in pregnancy in order to prevent some possible complications such as pain originating from the passage of stone particles and possible risk of preterm delivery (16). In Rana’s survey, pneumatic lithotripsy has been shown to be a safe, definite, and effective method for treatment of resistant-to-medical treatment stones in pregnant women (17).

Although there are some reports regarding utilization of out-of-body lithotripsy (26) and percutaneous lithotripsy (9, 27, 28) with or without using X-ray, ESWL and PCNL yet these methods are still contraindicated in pregnancy according to their probable risk for fetus and their usage is limited to post-delivery period.

Conclusion: Pneumatic lithotripsy can be a safe and effective method for urolithiasis in pregnancy. Of course, more research is needed to prove this as the standard method in pregnancy urolithiasis.
